# TRIM21通过与ZSWIM1相互作用调节肺腺癌细胞的增殖和迁移

**DOI:** 10.3779/j.issn.1009-3419.2024.101.13

**Published:** 2024-05-20

**Authors:** CHEN Luxuan, LIAN Qionghua, ZHANG Gui, WU Jiayao, ZENG Guandi, GAO Xuejuan

**Affiliations:** ^1^510632 广州，暨南大学肿瘤分子生物学教育部重点实验室，生物活性药物分子与成药性优化全国重点实验室，暨南大学生命与健康工程研究院（陈露璇，连琼华，章贵，吴嘉耀，曾观娣，高学娟）; ^1^MOE Key Laboratory of Tumor Molecular Biology and State Key Laboratory of Bioactive Molecules and Druggability Assessment, Institute of Life and Health Engineering, Jinan University, Guangzhou 510632, China; ^2^510317 广州，暨南大学附属广东省第二人民医院，传统医学与运动伤害康复研究所（陈露璇）; ^2^Guangdong Traditional Medical and Sports Injury Rehabilitation Research Institute, Affiliated Guangdong Second Provincial General Hospital of Jinan University, Guangzhou 510317, China

**Keywords:** 肺肿瘤, TRIM21, ZSWIM1, 增殖, 迁移, Lung neoplasms, TRIM21, ZSWIM1, Proliferation, Migration

## Abstract

**背景与目的:**

肺腺癌（lung adenocarcinoma, LUAD）是一种致病率和死亡率都极高的癌症，尽管现代医学的治疗手段在不断进步，但患者的5年生存率仍不高，因此我们想通过探究LUAD发生发展的分子机制进而鉴定新的治疗靶标。我们前期的研究报道了ZSWIM1（zinc finger SWIM-type containing 1）是一个促进LUAD细胞增殖、迁移、侵袭的新蛋白。本研究将着重探究E3泛素连接酶TRIM21（tripartite-motif protein 21）对ZSWIM1在LUAD细胞中促增殖、迁移功能的影响。

**方法:**

利用蛋白免疫共沉淀技术（co-immunoprecipitation, Co-IP）和免疫荧光技术（immunofluorescence, IF）验证TRIM21和ZSWIM1之间的相互作用和共定位；利用MTT和Transwell实验检测TRIM21及TRIM21、ZSWIM1协同对LUAD增殖、迁移的影响；利用蛋白印迹实验（Western blot, WB）检测TRIM21和ZSWIM1对LUAD细胞上皮间充质转化（epithelial-mesenchymal transition, EMT）标志物表达的影响；利用Co-IP联合WB检测TRIM21对ZSWIM1泛素化的影响。

**结果:**

TRIM21与ZSWIM1存在相互作用和共定位。TRIM21的上调可以抑制LUAD细胞的增殖和迁移。过表达TRIM21能够降低ZSWIM1对LUAD细胞增殖、迁移和侵袭的促进作用，并逆转ZSWIM1对E-cadherin和Vimentin表达的影响。敲低TRIM21则可以增强ZSWIM1对LUAD细胞增殖和迁移的促进作用。机制方面，我们发现过表达TRIM21可明显增强ZSWIM1的泛素化水平，下调ZSWIM1的蛋白表达量。

**结论:**

TRIM21通过结合并促进ZSWIM1的泛素化，进而降低ZSWIM1的蛋白表达，这抑制了ZSWIM1对LUAD细胞增殖、迁移、侵袭表型的促进作用。

## Abstract (en)

**Background and objective:**

Lung adenocarcinoma (LUAD) is a highly morbid and fatal cancer. Despite advancements in modern medical treatment, the 5-year survival rate of patients remains suboptimal. Our previous study revealed that zinc finger SWIM-type containing 1 (ZSWIM1), a novel protein, promotes the proliferation, migration, and invasion of LUAD cells. The aim of this study is to investigate the impact of E3 ubiquitin ligase tripartite motif protein 21 (TRIM21) on ZSWIM1-mediated cell proliferation and migration.

**Methods:**

The interaction and co-localization between TRIM21 and ZSWIM1 were verified using co-immunoprecipitation (Co-IP) and immunofluorescence (IF). The effects of TRIM21 and ZSWIM1 on the proliferation and migration of LUAD cells were assessed through MTT and Transwell assays, respectively. Western blot (WB) analysis was conducted to evaluate the impact of TRIM21 and ZSWIM1 on the expression of epithelial-mesenchymal transition (EMT) markers in LUAD cells. The influence of TRIM21 on the ubiquitination of ZSWIM1 was examined using Co-IP combined with WB.

**Results:**

TRIM21 was found to interact and co-localize with ZSWIM1. Overexpression of TRIM21 inhibited the proliferation and migration of LUAD cells. Overexpression of TRIM21 reduced the promoting effect of ZSWIM1 on the proliferation, migration, and invasion of lung adenocarcinoma cells, and reversed the impact of ZSWIM1 on the expression of E-cadherin and Vimentin. Conversely, knockdown of TRIM21 further enhanced the promoting effect of ZSWIM1 on the proliferation and migration of LUAD cells. Mechanistically, we observed that overexpression of TRIM21 significantly enhanced the ubiquitination level of ZSWIM1, leading to a decrease in ZSWIM1 protein expression.

**Conclusion:**

TRIM21 binds to and promotes the ubiquitination of ZSWIM1, resulting in reduced protein expression of ZSWIM1, which leads to the inhibition of ZSWIM1-mediated promotion of proliferation, migration, and invasion in LUAD cells.

肺癌是世界上发病率和死亡率最高的恶性肿瘤之一^[1]^，主要分为非小细胞肺癌（non-small cell lung cancer, NSCLC）和小细胞肺癌（small cell lung cancer, SCLC）^[2]^。其中，肺腺癌（lung adenocarcinoma, LUAD）是NSCLC最常见的类型^[1]^。随着医学技术的发展，对肺癌患者的治疗取得了一定的进展。然而，由于多数患者在确诊时已处于癌症晚期^[2]^，治疗难度大且效果不佳。因此，迫切需要深入了解LUAD发病的分子机制，以便寻找和确认新的LUAD诊疗标志物。

ZSWIM1（zinc finger SWIM-type containing 1）位于人类基因组20号染色体上，其编码的蛋白质含有一个锌指结构域和一个SWIM结构域。目前已经发现SWIM结构域存在于多种生物体中，如古细菌和哺乳动物^[3]^。其中MEKK1的SWIM结构域可特异性结合c-Jun蛋白的DNA结合结构域^[4]^。研究^[4]^发现，ZSWIM的表达上调对于T细胞尤其是CD4^+^辅助性T淋巴细胞的激活和分化具有重要作用。我们的前期研究^[5]^报道显示ZSWIM1可促进LUAD细胞的增殖和转移能力。然而，ZSWIM1具体的作用机制尚未明晰，有待进一步研究。在我们前期鉴定ZSWIM1的相互作用组学实验中，E3连接酶TRIM21引起我们的特别关注。

TRIM21（tripartite motif protein 21）是TRIM家族的成员之一，由一个RING结构域、一个B-box、一个coiled-coil和一个PRYSPRY 结构域组成。其中，RING结构域赋予TRIM21具有E3泛素连接酶的活性^[6,7]^。TRIM21在细胞核与细胞质中均有表达，它在不同肿瘤发生和发展过程中起着促进或抑制的作用^[7]^。在肝癌^[8]^、甲状腺癌^[9]^、胰腺癌^[10]^等癌症中，上调TRIM21的表达可促进癌细胞的增殖和耐药性；而在胃癌^[11]^、卵巢癌^[12]^、乳腺癌^[13]^等癌症中，上调TRIM21的表达则可抑制癌细胞的增殖和迁移。另外， TRIM21还广泛参与细胞分化、凋亡调节、先天免疫等生物过程。

在本研究中，我们将验证ZSWIM1与TRIM21之间的互相作用，及二者对LUAD增殖、迁移和侵袭的影响，以期进一步揭示LUAD的致病机制。

## 1 资料与方法

### 1.1 实验仪器及试剂

生物安全柜（HFsafe-CY）购于上海力康生物医疗科技有限公司；CO_2_细胞培养箱（MCO-20AIC）购于日本三洋公司；电泳仪及电泳转膜系统（Mini-PROTEAN Tetra Cell Systems）购于美国Bio-Rad公司；化学发光/荧光成像系统（Tanon 4600）购于上海天能科技有限公司；激光共聚焦显微镜LSM880购于德国ZEISS有限公司；酶标仪购于美国Bio-Tek公司；结晶紫粉末购于广州斯佳生物公司；爬片及载玻片（80346-0910）购于江苏世泰实验器材有限公司；小室购于美国Corning公司；MG132购于美国Selleck生物科技有限公司；DOX（ST039A）及BCA蛋白浓度测定试剂盒（P0009）购于上海碧云天生物科技公司；ZSWIM1、Ubiquitin抗体购于美国Santa Cruz公司；TRIM21、GAPDH、FLAG、Vimentin、E-cadherin抗体购于武汉三鹰生物技术有限公司。Vigfect购于北京威格拉斯生物技术有限公司，Lipo2000购于美国Invitrogen公司，Opti-MEM购于美国Gibco公司。

### 1.2 实验方法

#### 1.2.1 细胞培养及脂质体转染

人LUAD细胞系H1299和人293T细胞完全培养基：DMEM培养基+10% FBS；培养条件：5% CO_2_、37 ^o^C恒温培养箱。当细胞密度达到85%-90%时进行细胞传代并按实验需求将细胞铺进孔板中。

在6孔板中瞬转质粒（HA-Vector/TRIM21/Ub、FLAG-Vector/TRIM21），需满足细胞密度达到70%-80%，每个孔加入2 μg的质粒、1 μL的VigFect，需提前加入至无血清的培养基中，室温静置20 min，再加入细胞板中；4-6 h后换成完全培养基，培养48-72 h进行后续实验。

在293T细胞中转染带有目的基因的质粒（PTSB02-Vector/ZSWIM1-FLAG、H125-Vector/TRIM21）及病毒包装/包膜质粒（pSPAX2/pMD2.G），48 h后收集病毒液转染目的细胞，随后用嘌呤霉素对细胞进行筛选，得到稳定表达目的基因的细胞株。每次实验前需用DOX（工作浓度为10 μg/mL）处理转染H125质粒的细胞24 h，诱导目的蛋白表达。

在6孔板中转染小干扰RNA（small interfering RNA, siRNA），需满足细胞密度达到40%-50%，每孔加入100 nmol/L的siRNA片段、3.5 μL的Lipo2000，需提前在Opti-MEM中混匀静置20 min，后续步骤同上。其中的TRIM21的siRNA序列为片段1：5’-GCAGGAGUUGGCUGAGAAGTT-3’；片段2：5’-GGACAATTTGGTTGTGGAA-3’；片段3：5’-GGAATGCATCTCTCAGGTT-3’。

#### 1.2.2 蛋白印迹实验（Western blot, WB）

向10 cm细胞培养皿中加入相应体积的EBC Buffer（已加PI蛋白酶、PMSF、Na_3_VO_4_、NaF共4种抑制剂，且EBC和抑制剂的比例为100:1），冰上裂解30 min，4 ^o^C离心30 min、12,000 rpm，收集蛋白上清液。BCA法测定蛋白浓度，以每孔相同的质量上样，在10%-12%的SDS-PAGE凝胶中分离蛋白，在低温条件下进行转膜（采用PVDF膜，电湿转）。随后在室温的条件下封闭2 h（封闭液为1×TBST含5%脱脂奶粉），再分别用anti-FLAG（1:2000稀释）、anti-TRIM21（1:1000稀释）、anti-GAPDH（1:2000稀释）、anti-E-cadherin（1:1000稀释）、anti-Vimentin（1:1000稀释）、anti-ZSWIM1（1:1000稀释）、anti-Ub（1:1000稀释），4 ^o^C过夜孵育，第2天用1×TBST清洗PVDF膜，10 min/次，共3次。用二抗工作液（1:4000稀释）室温孵育2 h，结束后同样用1×TBST清洗3遍，按1:1配制ECL发光液，在成像仪显影。

#### 1.2.3 免疫荧光实验（immunofluorescence, IF）

对消化下来的细胞悬液进行计数，每孔（2-3）×10^4^个细胞于24孔板中，待贴壁后用PBS清洗细胞，再用固定液（恢复室温的4%多聚甲醛）室温静置30 min；用2 mg/mL Glycine缓冲液清洗1遍，PBS清洗3遍后加入含0.2% TritonX-100的PBS，常温静置透化10 min；结束后PBS清洗3遍，加入10%的山羊血清NGS（用PBS配制），常温封闭1 h；再加入一抗工作液（一抗:NGS:PBS=1:2:100）4 ^o^C孵育过夜；第二天用清洗缓冲液（用含1%的牛血清白蛋白的PBS配制）清洗5次；每孔加入对应的二抗工作液（二抗:NGS:PBS=1:2:100），室温避光孵育1.5 h，继续用清洗缓冲液清洗5次；最后用稀释好（用PBS按1:1000稀释）的DAPI室温染色10 min，清洗5次后，在载玻片上滴一滴抗荧光淬灭封片液，将爬片有细胞的一面与其接触，静置晾干，在激光共聚焦显微镜下拍照。

#### 1.2.4 蛋白免疫共沉淀实验（co-immunoprecipitation, Co-IP）

收集10 cm细胞培养皿90%密度以上的细胞，加入相应体积的EBC Buffer（含4种蛋白酶抑制剂）冰上裂解30 min，12,000 rpm、4 ^o^C离心30 min，收集蛋白上清液。每管中加入2 μg的IgG，在4 ^o^C旋转仪上结合30 min每管再加入40 μL的Protein A/G Agarose珠子在4 ^o^C旋转仪上结合30 min，4 ^o^C、3000 rpm离心2 min，收集上清，向其中加入40 μL的Protein A/G Agarose珠子在4 ^o^C旋转仪上结合60 min，4 ^o^C、3000 rpm离心2 min，收集上清，利用BCA法测蛋白浓度。根据蛋白质量分成3组：Input组、IgG组、IP组，分别加入2 μg的IgG和2 μg对应的抗体。Input组加好1×SDS Loading后放到沸水中煮10 min，冷却后暂存-80 ^o^C。其余两组在4 ^o^C旋转仪上结合过夜，再加入40 μL的Protein A/G Agarose珠子于4 ^o^C旋转仪上结合4-6 h离心保留珠子沉淀，并用EBC Buffer清洗5次，第5次尽量吸干上清，随后每管加入40 μL 1×SDS Loading，沸水煮10 min。最后，对3组样品进行WB实验检测。

#### 1.2.5 Transwell实验

对消化下来的细胞悬液进行计数，每孔8×10^3^个细胞。在24孔板下室加入700-800 μL完全培养基，上室加入细胞悬液，下室加入完全培养基。需要做侵袭的组，将稀释好的基质胶加入到上室中（基质胶:无血清培养基=1:10-1:20），置于细胞培养箱中使其凝固。培养一段时间后将小室夹出，于4%多聚甲醛中静置30 min，再置于0.1%的结晶紫染料中，15 min后取出用湿棉签处理内层未迁移的细胞及残余结晶紫，自然风干后，在显微镜下选取上下左中右5个视野拍照记录，统计数据。

#### 1.2.6 MTT实验

对消化下来的细胞悬液进行计数，每孔1000个细胞/200 μL培养基，每组设置5个复孔，边混匀边打入96孔板中，待贴壁后即可开始测量。用完全培养基将5 mg/mL的MTT原液稀释十倍后，200 μL稀释后的MTT工作液依次加入到去除培养基的孔中，该操作需要全程避光，将96孔板放回CO_2_细胞培养箱中，4 h后再取出弃掉工作液，加入150 μL DMSO，10 min后使用酶标仪在570 nm波长下测定吸光值。根据实验需求设置测定次数。

### 1.3 统计学方法

本课题每个实验均进行至少3次生物学重复，各指标以Mean±SD表示结果，采用GraphPad Prism绘图，采用t检验或单因素方差分析等统计学方法对实验结果进行统计分析，P<0.05为有统计学差异。

## 2 结果

### 2.1 H1299细胞中TRIM21与ZSWIM1存在相互作用

TRIM21是我们前期利用IP-MS技术鉴定到的ZSWIM1蛋白的一个潜在相互作用伙伴^[4]^。为了验证它们之间的相互作用，我们在稳定过表达FLAG-ZSWIM1（OE-FLAG-ZSWIM1）的H1299细胞中转入HA-TRIM21质粒，使用FLAG抗体进行Co-IP实验。结果显示在过表达ZSWIM1和TRIM21的细胞中鉴定到二者的相互作用（图1A）。接着，我们利用IF实验检测ZSWIM1和TRIM21的共定位情况。结果显示，H1299细胞中，ZSWIM1主要分布于细胞质及细胞膜附近，TRIM21在细胞质与细胞核均有分布，二者的共定位主要发生在细胞膜附近（图1B）。我们的结果提示TRIM21与ZSWIM1存在相互作用与共定位。

**图 1:**
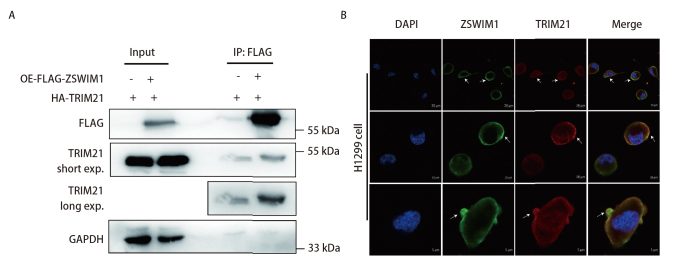
在肺腺癌细胞中TRIM21与ZSWIM1存在相互作用。A：在稳定过表达FLAG-ZSWIM1的细胞中瞬转HA-TRIM21，利用FLAG抗体进行Co-IP实验；B：在H1299细胞中对内源的ZSWIM1和TRIM21进行染色并在共聚焦显微镜下观察，其中ZSWIM1为绿色，TRIM21为红色，细胞核为蓝色，箭头：TRIM21和ZSWIM1共定位的位置。比例尺依次为20、10和5 μm。

### 2.2 TRIM21可抑制H1299的增殖、迁移能力

我们通过Co-IP和IF实验确认TRIM21与ZSWIM1存在相互作用，为了进一步了解TRIM21在LUAD细胞中的功能，我们构建了稳定过表达TRIM21的H1299细胞株，在加入DOX诱导后利用WB检测过表达情况（图2A），结果显示TRIM21的蛋白量明显增加。再利用MTT及Transwell实验检测H1299的增殖（图2B）及迁移能力（图2C）。MTT实验结果显示过表达TRIM21后H1299细胞的增殖能力被抑制（P<0.01），迁移实验结果显示过表达TRIM21后细胞的迁移能力减弱（P<0.01）（图2D）。接着我们在H1299中敲低TIRM21，利用WB检测敲低情况，结果显示TRIM21的3条siRNA都能有效干扰TRIM21的表达（图2E），我们选取了前两条完成接下来的实验。我们利用MTT及Transwell实验发现敲低TRIM21后LUAD细胞的增殖（图2F）及迁移能力（图2G、2H）增强。以上结果提示TRIM21可以抑制LUAD细胞的增殖和迁移能力。

**图 2:**
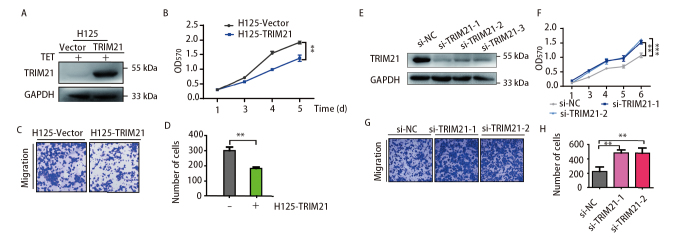
TRIM21抑制肺腺癌细胞中的增殖、迁移功能。A：利用WB实验检测稳定过表达TRIM21的H1299细胞株中TRIM21的表达情况；B：在稳定过表达TRIM21的H1299细胞株中，利用MTT实验检测细胞的增殖能力（n=3, ^**^P<0.01）；C、D：利用Transwell实验检测过表达TRIM21细胞的迁移能力（n=3, ^**^P<0.01）；E：在H1299中瞬转TRIM21的3条siRNA片段，利用WB实验检测TRIM21的表达情况；F：在H1299中敲低TRIM21，利用MTT实验检测细胞的增殖能力（n=3, ^**^P<0.01, ^***^P<0.001）；G、H：在H1299中敲低TRIM21，利用Transwell实验检测细胞的迁移能力（n=3, ^**^P<0.01）。

### 2.3 敲低TRIM21可增强ZSWIM1对H1299细胞增殖和迁移的促进作用

我们发现TRIM21抑制LUAD的增殖与迁移且TRIM21又与ZSWIM1具有相互作用，那TIRM21对ZSWIM1在LUAD中的功能有何影响呢?在我们之前的报道中，已经证明ZSWIM1蛋白对LUAD细胞增殖、迁移和侵袭能力的促进作用^[4]^。

于是我们在过表达ZSWIM1的细胞中敲低TRIM21，检测细胞增殖和迁移表型的变化。结果表明TRIM21的2条siRNA片段均能在过表达ZSWIM1的细胞中有效敲低TRIM21的表达（图3A）。MTT实验表明干扰TRIM21表达后，ZSWIM1的促增殖功能进一步增强（图3B），且具有统计学差异（片段1，P<0.001；片段2，P<0.01）。

**图 3:**
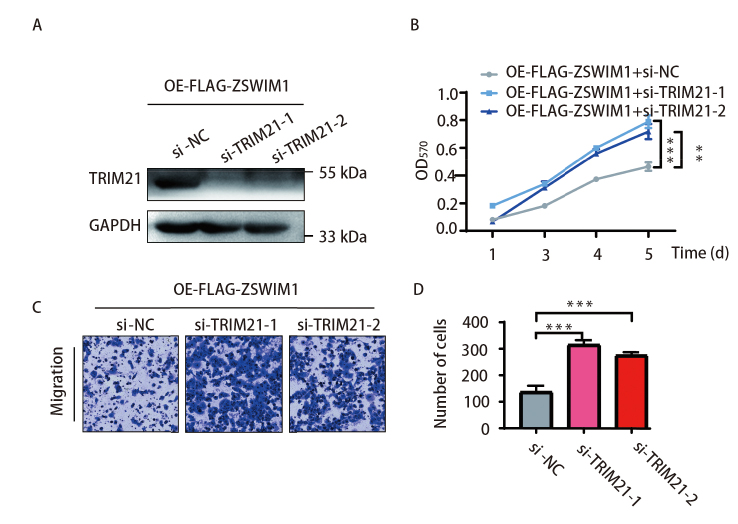
干扰TRIM21的表达可增强ZSWIM1诱导的H1299细胞增殖、迁移。在稳定过表达ZSWIM1的H1299细胞中敲低TRIM21，A：利用WB实验检测TRIM21的表达情况；B：利用MTT实验检测细胞的增殖能力（n=3, ^**^P<0.01, ^***^P<0.001）；C、D：利用Transwell实验检测细胞的迁移能力（n=3, ^***^P<0.001）。

同时，Transwell实验结果显示，在过表达ZSWIM1的基础上敲低TRIM21，则进一步增强H1299细胞的迁移能力（图3C、3D）（P<0.001）。结果提示敲低TRIM21使得ZSWIM1对LUAD细胞的增殖、迁移和侵袭的促进作用进一步增强。

### 2.4 过表达TRIM21可下调ZSWIM1对H1299细胞增殖、迁移、侵袭的促进作用

我们验证TRIM21与ZSWIM1的相互作用是否协同调节LUAD细胞的增殖、迁移和侵袭能力。我们在稳定过表达ZSWIM1的细胞中转染HA-TRIM21或者HA-Vector，利用Transwell实验发现过表达TRIM21后，细胞的迁移（P<0.01）和侵袭能力（P<0.001）明显下降（图4A、4B）。 同样，MTT实验的结果显示，在OE-FLAG-ZSWIM1细胞中过表达TRIM21后，细胞的增殖能力显著下调（图4C）。

**图 4:**
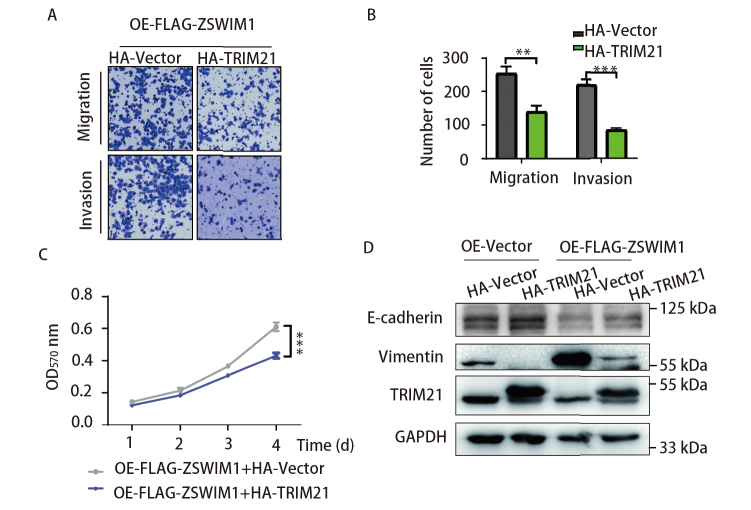
过表达TRIM21可减缓ZSWIM1对H1299细胞增殖、迁移、侵袭的促进作用。A、B：在稳定过表达ZSWIM1（OE-FLAG-ZSWIM1）的H1299细胞中瞬转HA-TRIM21，利用Transwell实验检测细胞的迁移、侵袭能力（n=3, ^**^P<0.01, ^***^P<0.001）；C：在稳定过表达ZSWIM1的H1299细胞中瞬转HA-TRIM21，利用MTT实验检测细胞的增殖能力（n=3, ^***^P<0.001）；D：同时在H1299细胞中过表达ZSWIM1和TRIM21，利用WB检测EMT标志物E-cadherin、Vimentin。

在多种纤维化疾病、慢性炎症、肿瘤细胞的转移中，细胞上皮间充质转化（epithelial-mesenchymal transition, EMT）发挥着重要作用，通过EMT癌细胞获得了迁移与侵袭等间质表型^[14]^。EMT的主要特征是E-钙黏蛋白（E-cadherin）的丧失、间充质标记物（如波形蛋白、Vimentin）的增加。我们检测了二者的相互作用对LUAD细胞EMT过程的协同影响。WB的结果显示，过表达ZSWIM1下调E-cadherin的表达，上调Vimentin的表达；而同时过表达TRIM21，则逆转了ZSWIM1对E-cadherin表达的下调，以及对Vimentin表达的上调（图4D）。

这些结果说明TRIM21能够抑制ZSWIM1在LUAD细胞中的促癌功能。

### 2.5 TRIM21通过泛素化途径降解ZSWIM1

为了探寻TRIM21对ZSWIM1功能的影响机制，考虑到TRIM21具有E3连接酶的功能，我们检测了TRIM21对ZSWIM1蛋白表达的影响，结果显示在过表达TRIM21的情况下，ZSWIM1的蛋白表达量明显下降（图5A）。于是我们在过表达ZSWIM1和TRIM21的基础上，转染泛素分子，并用MG132处理来抑制26S蛋白酶体复合物的蛋白水解活性，然后通过Co-IP技术检测ZSWIM1的泛素化情况。结果显示，过表达TRIM21明显增强ZSWIM1的泛素化（图5B）。这些结果提示TRIM21对ZSWIM1蛋白表达量的下调可能是通过增强ZSWIM1的泛素化实现的。

**图 5:**
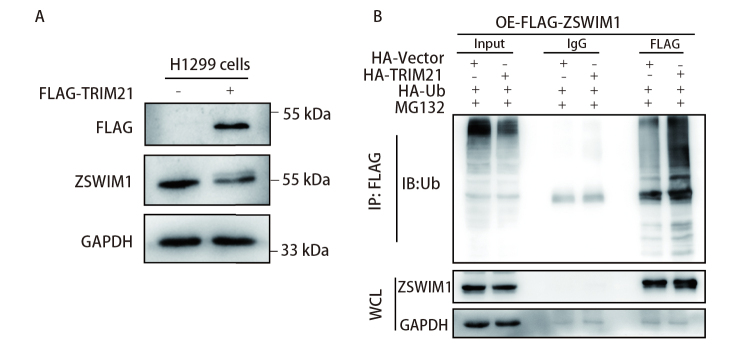
过表达TRIM21可增强ZSWIM1的泛素化。A：在H1299细胞中瞬转FLAG-TRIM21，利用WB检测ZSWIM1蛋白的表达情况；B：在稳定过表达ZSWIM1的H1299细胞中瞬转HA-TRIM21和HA-Ub，并用MG132（20 μmol/L）处理细胞，利用Co-IP技术检测ZSWIM1的泛素化情况。

## 3 讨论

LUAD细胞具有高浸润性和转移性，中晚期患者治疗难度大、治疗预后差^[2]^。因此，急需了解LUAD发生发展的分子机制进而鉴定新的治疗靶标。本研究主要探索了TRIM21对ZSWIM1在LUAD中功能的影响。首先，我们通过Co-IP和IF实验证实了TRIM21与ZSWIM1之间的相互作用关系。通过过表达或敲低TRIM21验证了TRIM21在LUAD细胞中的抑癌功能。接着，在稳定过表达ZSWIM1的H1299细胞株，我们进行了TRIM21的过表达或干扰实验，并通过MTT和Transwell实验验证了TRIM21可以逆转ZSWIM1对LUAD细胞增殖、迁移和侵袭的促进作用。作用机制方面，我们发现TRIM21可能通过泛素化降解ZSWIM1，使得ZSWIM1的蛋白含量降低，这有待进一步的深入研究。

我们的前期研究表明，ZSWIM1在LUAD组织中表达上调，并且过表达ZSWIM1显著促进了LUAD细胞的增殖、迁移、侵袭和EMT；机制方面，ZSWIM1与丝氨酸/苏氨酸激酶38（serine threonine kinase 38, STK38）相互作用，并促进MEKK2/ERK1/2通路的激活^[5]^。此外，在TISIDB的分析中发现，ZSWIM1在子宫内膜癌中的表达与调节性T细胞呈负相关性^[15]^，并与肿瘤浸润性淋巴细胞有一定关系。在前期研究中，我们通过IP-MS质谱技术鉴定了ZSWIM1相互作用组学^[4]^。ZSWIM1含有一个SWIM结构，是一种类锌指状结构域，能够在不同的条件下介导与特定蛋白质/DNA的相互作用^[16]^。

IF实验显示ZSWIM1与TRIM21主要共定位在细胞膜周围。当TRIM21与ZSWIM1结合后，ZSWIM1在细胞增殖、迁移、侵袭方面的功能被减弱。

TRIM21蛋白的功能与抗病毒反应和自身免疫性疾病密切相关^[17,18]^。TRIM21参与肿瘤的发生发展，并且其泛素化底物多为与肿瘤发生和肿瘤治疗相关的关键分子。例如，在肝癌中，β-连环素（β-catenin）的表达上调与肝癌的发生发展密切相关^[19]^。TRIM21与β-catenin结合并发挥E3泛素连接酶的作用，将更多的泛素分子结合到β-catenin上，通过泛素化途径降解β-catenin^[20]^。在肾癌中，TRIM21通过泛素化介导了缺氧诱导因子1亚基α（hypoxia-inducible factor 1 alpha, HIF-1α）的降解，HIF-1α的表达下调抑制了糖酵解，使得肾癌细胞的转移、增殖能力被抑制^[21]^。在人胶质母细胞瘤中，TRIM21介导PFK1血小板异构体（glycolysis phosphofructokinase platelet, PFKP）多泛素化和降解，抑制了PFK1对细胞增殖和脑肿瘤生长的促进作用^[22]^。在结直肠癌中，MICAL样2蛋白（molecule interacting with CasL-like protein 2, MICALL2）在结直肠癌组织中上调，TRIM21泛素化降解MICALL2后，抑制了MICALL2的促癌作用^[23]^。TRIM21蛋白N端的RING结构域具有E3泛素连接酶的活性^[7]^。有研究^[24]^构建了缺乏E3泛素连接酶活性的TRIM21-ΔRING截短体质粒，在HEK293T细胞中过表达TRIM21组泛素化降解了UBE2M（ubiquitin-conjugating enzyme E2M）而不是TRIM21-Δ-RING截短体组，进而抑制UBE2M诱导产生IFN-I。在本研究中，我们确认了TRIM21作为E3连接酶可以促进ZSWIM1的泛素化增加。然而，关于TRIM21如何调节ZSWIM1的泛素化仍需要进一步的实验进行探索。

综上所述，我们发现在LUAD中，TRIM21可能通过泛素化降解ZSWIM1，从而调节ZSWIM1的功能。单独过表达ZSWIM1可以促进LUAD细胞的增殖、迁移、侵袭和EMT过程^[5]^。然而，当TRIM21的表达量增加时，可以抑制ZSWIM1的促癌功能。我们的研究为进一步深入理解TRIM21、ZSWIM1在LUAD增殖和转移过程中的调节作用提供了一定的理论基础。
